# Diagnosis, management and treatment of the Alport syndrome – 2024 guideline on behalf of ERKNet, ERA and ESPN

**DOI:** 10.1093/ndt/gfae265

**Published:** 2024-12-02

**Authors:** Roser Torra, Beata Lipska-Zietkiewicz, Frederic Acke, Corinne Antignac, Jan Ulrich Becker, Emilie Cornec-Le Gall, Albertien M van Eerde, Nicolas Feltgen, Rossella Ferrari, Daniel P Gale, Susie Gear, Oliver Gross, Stefanie Haeberle, Laurence Heidet, Rachel Lennon, Laura Massella, Kristina Pfau, Maria del Prado Venegas Pizarro, Rezan Topaloglu, Tanja Wlodkowski, Heidi Zealey, ERKNet, ERA Genes&Kidney and ESPN Inherited renal disorders working group, Oana Ailioaie, Marina Aksenova, Peter Barany, Moumita Barua, Elisa Benetti, Lisa Bonebrake, Olivier Bonny, Antonia Bouts, Olivia Boyer, Gianluca Caridi, Cristina Castro-Alonso, Kathleen Claes, Peter Conlon, George Claudiu Costea, Stéphane Decramer, Constantinos Deltas, Erol Demir, Nathalie Demoulin, Mark Eijgelsheim, Francesco Emma, Frances Flinter, Monica Furlano, Danica Galešić Ljubanović, Valentine Gillion, Ana Marta Gomes, Dieter Haffner, Julia Hoefele, Svetlana Jovicic Pavlovic, Clifford Kashtan, Stefan Kohl, Martin Konrad, Matjaž Kopač, Sandrine Lemoine, Max Christoph Liebau, Francesca Lugani, Alvaro Madrid, Andrew Mallett, Antonio Mastrangelo, Anamarija Meglič, Esther Meijer, Jeffrey Miner, Sevgı Mır, Kar Hui Ng, João Paulo Oliveira, Maria Vanessa Perez Gomez, Anna Maria Pinto, Ann Raes, Michelle Rheault, Judy Savige, Christoph Schwarz, Angel Manuel Sevillano Prieto, Ekamol Tantisattamo, Velibor Tasic, Kálmán Tory, Neil Turner, Andre Weinstock, Izabela Zakrocka

**Affiliations:** Nephrology Department, Fundació Puigvert, Institut de Recerca Sant Pau (IR-Sant Pau), Departament de Medicina, Universitat Autònoma de Barcelona, RICORS2040, Barcelona, Spain; Clinical Genetics Unit, Department of Biology and Medical Genetics, Medical University of Gdańsk, Gdańsk, Poland; Rare Diseases Centre, Medical University of Gdańsk, Gdańsk, Poland; Department of Otorhinolaryngology, 1P1, Ghent University / Ghent University Hospital, Ghent, Belgium; Laboratory of Hereditary Kidney Diseases, Inserm UMR 1163, Imagine Institute, Université Paris Cite, Paris, France; Institute of Pathology, University Hospital of Cologne, Cologne, Germany; University Brest, Inserm, UMR 1078, GGB, CHU Brest, Centre de référence MARHEA, filière ORKID, Brest, France; Department of Genetics, University Medical Center Utrecht, Utrecht, The Netherlands; Department of Ophthalmology, University Hospital Basel, Basel, Switzerland; Italian Association of Alport Syndrome, (ASAL) ERKNet ePAG Castellarano, Emilia-Romaña, Italia; Centre for Kidney and Bladder Health, University College London, London, UK; International Alport Syndrome Alliance and Alport UK, ERKNet ePAG, Cirencester, Gloucestershire, UK; Department of Nephrology and Rheumatology, University Medical Center Göttingen, Göttingen, Germany; Division of Paediatric Nephrology, Center for Paediatrics and Adolescent Medicine, University Hospital, Heidelberg, Germany; Néphrologie Pédiatrique, Centre de Référence MARHEA, APHP, Hôpital universitaire Necker-Enfants Malades, Paris, France; Department of Paediatric Nephrology, Royal Manchester Children's Hospital, Manchester University Hospitals NHS Foundation Trust, Manchester Academic Health Science Centre, Manchester, UK; Division of Nephrology, Bambino Gesù Children's Hospital, IRCCS, Rome, Italy; Institute of Molecular and Clinical Ophthalmology Basel, Basel, Switzerland; Otolaryngology-Head and Neck Surgery, Hospital de Sant Pau i la Santa Creu, Instituto de Investigaciones Biomédicas Sant Pau, Universitat Autónoma de Barcelona, Barcelona, Spain; Division of Pediatric Nephrology, Hacettepe University Faculty of Medicine, Ankara, Turkey; Division of Paediatric Nephrology, Center for Paediatrics and Adolescent Medicine, University Hospital, Heidelberg, Germany; International Alport Syndrome Alliance and Alport UK, ERKNet ePAG, Cirencester, Gloucestershire, UK

**Keywords:** Alport syndrome, collagen IV, glomerular basement membrane, haematuria, *COL4A3/4/5*

## Abstract

Glomerular nephropathy resulting from the genetic defects in *COL4A3/4/5* genes including the classical Alport syndrome is the second most common hereditary kidney disease characterized by persistent haematuria progressing to the need for kidney replacement therapy, frequently associated with sensorineural deafness, and occasionally with ocular anomalies.

Diagnosis and management of *COL4A3/4/5* glomerulopathy is a great challenge due to its phenotypic heterogeneity, multiple modes of inheritance, variable expressivity, and disease penetrance of individual variants as well as imperfect prognostic and progression factors and scarce and limited clinical trials, especially in children.

As a joint initiative of the European Rare Kidney disease reference Network (ERKNet), European Renal Association (ERA Genes&Kidney), and European Society for Paediatric Nephrology (ESPN) Inherited renal disorders working group, a team of experts including adult and paediatric nephrologists, kidney geneticists, audiologists, ophthalmologists, and a kidney pathologist were

selected to perform a systematic literature review on 21 clinically relevant PICO (Patient or Population covered, Intervention, Comparator, Outcome) questions. The experts formulated recommendations and formally graded them at a consensus meeting with input from patient representatives and a voting panel of nephrologists representing all regions of the world.

Genetic diagnostics comprising joint analysis of *COL4A3/4/5* genes is already the key diagnostic test during the initial evaluation of an individual presenting with persistent haematuria, proteinuria, kidney failure of unknown origin, focal segmental sclerosis of unknown origin, and possibly cystic kidney disease. Early renin-angiotensin system blockade is the standard of care therapy; sodium-glucose cotransporter-2 inhibitors may be added in adults with proteinuria and chronic kidney disease. Relatives with heterozygous *COL4A3/4/5* variants should only be considered as the last possible resource for living kidney donation.

This guideline provides guidance for the diagnosis and management of individuals with pathogenic variants in *COL4A3/4/5* genes.

## INTRODUCTION

The focus of this guideline is to describe the current state-of-art in the diagnosis, management, and treatment of the disorders resulting from the genetic defects in *COL4A3/4/5* genes including the classical Alport syndrome (AS) (ORPHA 63). The document is a joint initiative of ERKNet, ERA Genes&Kidney, and ESPN Inherited renal disorders working group along with patient advocacy groups. There is an ongoing discussion on better defining the name for this entity [1]. The key clinical manifestation is persistent haematuria defined as present in two or more samples over a 6- to 12-month period. The classical triad of symptoms comprises familial progressive kidney disease with sensorineural hearing loss and ocular defects, although the penetrance and expressivity of these phenotypes is variable from isolated haematuria to early kidney failure (KF). Kidney biopsies may show various light microscopic abnormalities including normal appearances, focal segmental glomerulosclerosis, tubulointerstitial atrophy, and even IgA deposits mimicking IgA nephropathy; however, electron microscopic analysis typically reveals abnormalities, splitting, or thickening of the glomerular basement membrane (GBM) [2].

## MATERIALS AND METHODS

Eight working groups (WG) were established to examine different clinical aspects of AS (Definition/Epidemiology, Prognosis, Management, Management Ear, Management Eye, Treatment, Transplantation). The WGs defined a total of 21 PICO (Patient or Population covered, Intervention, Comparator, Outcome) research questions. A systematic literature search was conducted in PubMed based on relevant key words. Electronic records of the references retrieved by searches were stored using a reference management web application (Rayyan) [3]. For the initial screening process, at least two members of the writing committee scanned titles and abstracts to exclude irrelevant publications. Original articles for selected references were retrieved, and full-text screening was processed.

Initial statements were elaborated and discussed by each WG. Evidence grading was done according to the methodology of the American Academy of Pediatrics [4]. External voting groups (ERKNet WG for hereditary glomerulopathies, ESPN Inherited renal disorders working group, ERA Genes&Kidney, international experts, and Alport Alliance) were asked to provide the level of agreement via a Delphi survey. A minimum of 70% consensus was required for the final adoption of the recommendations.

## Q1: XLAS AND ARAS: WHAT ARE THE DIFFERENCES AMONG THEM AND WHAT IS THEIR PREVALENCE?

**1-Statement: XLAS is much more prevalent than ARAS and affects males and females differently.** (Grade A, strong)

AS (ORPHA 63) can be subdivided according to either the observed inheritance pattern or the underlying molecular defect into X-linked Alport syndrome (XLAS; ORPHA 88917), which is linked with pathogenic variants affecting the *COL4A5* gene on the X chromosome, and autosomal recessive Alport syndrome (ARAS; ORPHA 88919), linked with biallelic pathogenic variants affecting *COL4A3* or *COL4A4*, which are situated next to each other on chromosome 2 [5]. In exceptional cases the defect in *COL4A5* also affects part of the adjacent *COL4A6* gene leading to additional development of smooth muscle tumours (ORPHA 1018) [6].

AS is the second most common Mendelian cause of kidney disease. Estimates of the combined phenotype-based prevalence of XLAS and ARAS in historical literature vary, ranging from 1:5000 in Utah [7] to 1:17 000 in Sweden [8] and 1:53 000 in Finland [9], with the recessive form accounting for around 15% of families. More recently, analysis of population-based genome sequencing data in GnomAD showed that 1:2320 individuals harbour a predicted pathogenic *COL4A5* variant with the proportion being higher in those with European ancestry (1:1800) compared to those with East Asian (1:2310) or Ashkenazi Jewish (1:4961) ancestry [10]. The population frequency of a predicted pathogenic variant in *COL4A3* or in *COL4A4* was 0.41% and 0.42%, respectively. Homozygous pathogenic *COL4A3* or *COL4A4* are reported in GnomAD but it is not possible to infer compound heterozygosity in this dataset. However, calculation of the expected frequency of biallelic disease, inferred from the observed frequency of heterozygous pathogenic variants in either of these genes, was 1:88 866 under the assumption of independent occurrence of variants. This may underestimate the true frequency of autosomal recessive disease given the presence of founder pathogenic variants in certain populations [e.g. *COL4A4*: c.2906C>G; p.(Ser969Ter) in the UK] and assortative mating that is prevalent across human populations [11].

However, since not all individuals with qualifying variants in these population studies exhibit haematuria or other clinical evidence of AS, this may lead to an overestimation of the disease prevalence, which would need future studies addressing penetrance (see Q11).

## Q2: WHAT ARE THE PENETRANCE AND PREVALENCE OF HETEROZYGOUS *COL4A3/COL4A4* SINGLE PATHOGENIC VARIANTS?

**2-Recommendation: Single pathogenic variants in *COL4A3/COL4A4* are relatively frequent in the general population and therefore their presence should not be regarded as the only cause of the kidney disease and other/additional diagnoses (e.g. IgA nephropathy) should be considered.** (Grade B, strong)

The presence of one pathogenic or likely pathogenic (P/LP) allele of *COL4A3* or *COL4A4* is relatively common in the general population, occurring in 0.94% (or 1:106) of individuals in GnomAD, with the frequency ranging from 0.24% in Finnish to 1.58% in Latino populations [10]. While the presence of such a variant in heterozygosity can be regarded as a risk factor for having offspring with ARAS (which requires inheritance of the P/LP allele along with a defect in the other allele), the term ‘ARAS carrier’ is generally avoided to describe such individuals. This is because the presence of a monoallelic P/LP *COL4A3* or *COL4A4* variant is itself sufficient to cause clinical manifestations, with haematuria and microalbuminuria strongly associated in population-based genome sequencing studies [10, 12, 13]. The lifetime risk of clinically significant kidney impairment or KF attributable to heterozygosity for such variants remains less clear. While several studies have identified multigenerational families with clinically significant phenotypes such as focal segmental glomerulosclerosis, chronic kidney disease (CKD), and KF segregating with monoallelic P/LP *COL4A3* or *COL4A4* variants, and sequencing studies in unselected kidney disease cohorts recognize these variants as important contributors to kidney disease in the population [14, 15], the absolute risk of KF due to these genetic variants remains poorly understood. It is likely much lower than the 15% risk previously estimated from older family-based studies, which were likely influenced by ascertainment and reporting biases [16, 17]. Given that the prevalence of monoallelic P/LP *COL4A3* or *COL4A4* variants in the general population is close to 1%, the absolute risk of KF attributable to the presence of such a variant must be far smaller, with more recent estimates putting it below 3% [18]. Where kidney biopsies are performed in individuals with a monoallelic P/LP *COL4A3* or *COL4A4* variant, various light microscopic abnormalities have been reported, including normal appearances, minimal change disease, mesangioproliferative nephritis, membranous nephritis, focal segmental glomerulosclerosis, tubulointerstitial nephritis and atrophy, and even IgA deposits [19, 20]. However, electron microscopy typically reveals thinning of the GBM in most individuals. Other abnormalities, such as irregularities, areas of thickening, lamination, basket-weaving, and wrinkling, may also be present in some cases [16].

Due to the various ways of identifying patients with heterozygous P/LP *COL4A3* or *COL4A4* variants, several diagnostic labels are (or have been) used, including ‘thin basement membrane nephropathy’, ‘autosomal dominant Alport syndrome’, ‘Type IV collagen-associated kidney disease’, ‘Alport spectrum nephropathy’ and, historically, ‘benign familial haematuria’. There is a strong consensus among medical professionals that the term ‘benign’ should no longer be used. This decision stems from the understanding that, although the absolute risk of KF is modest, it is significantly higher than that of the general population [2, 16, 21]. Despite the current uncertainty over nomenclature there is an emerging appreciation of the high frequency, and hence clinical and epidemiological importance of heterozygous P/LP *COL4A3* or *COL4A4* variant-associated disease [22].

## Q3: IS THE TERM ‘DIGENIC’ JUSTIFIED IN AS?

**3-Statement: The presence of pathogenic variants in more than one of *COL4A3/4/5*, sometimes called ‘digenic’, may lead to an age at onset of KF that is intermediate between that reported for XLAS or ARAS and monoallelic autosomal disease.** (Grade D, expert opinon)

The term ‘digenic’ AS refers to the situation in which there are P/LP changes in two of the *COL4A3, COL4A4*, or *COL4A5* genes in an individual. This can be subdivided into (i) a change in *COL4A5* plus a change in one of either *COL4A3* or *COL4A4*, and (ii) a change in one copy of *COL4A3* and a change in one copy of *COL4A4* (which may be *in cis* or *in trans*) in an individual. In the latter situation the phase of the variants affects the risk to the offspring of inheriting digenic or monoallelic disease [23, 24].

It is likely that the severity of AS (measured by the median age at onset of KF) can be increased by digenic disease if the proportion of normal type IV collagen trimer is reduced compared to the monoallelic setting. For instance, in males with a single defective *COL4A5* allele all relevant trimers will be abnormal, so the presence of an additional *COL4A3* or *COL4A4* variant may not be clinically apparent. This is consistent with the lack of earlier onset of KF in 16 male individuals reported in the largest series to date [23]. In females with heterozygous pathogenic changes in both *COL4A5* and one of *COL4A3* or *COL4A4*, there will be a reduction in a fraction of normal trimers compared with females with a faulty copy of only *COL4A5*, so severity of disease is predicted to be greater. While current empirical evidence is weak, showing only a trend towards earlier onset proteinuria in 26 females with digenic disease in the largest series to date [23], future analyses with larger sample sizes may confirm this logical hypothesis.

Individuals of either sex with heterozygous pathogenic alleles of both *COL4A3* and *COL4A4* (autosomal digenic disease) are predicted to produce normal trimeric type IV collagen chains (i.e. that incorporate only wild-type alleles) in only 25% of the time, resulting in more severe disease than individuals with a single pathogenic variant in either *COL4A3* or *COL4A4*, where 50% of the trimers are predicted to be normal. Determining whether the variants are *in ci*s or *in trans* is important for genetic counselling as the risk to offspring is different for each situation. Analysis of a cohort of 32 individuals with autosomal digenic disease revealed a median age at onset of KF that was intermediate between that of ARAS [23, 25], and monoallelic disease [17]. Given that the frequency of heterozygous pathogenic variants in *COL4A3* or *COL4A4* is approximately 1%, this is the proportion of individuals with XLAS who are predicted to harbour an additional autosomal variant, assuming independent inheritance of variants in the relevant genes. This estimate is supported by analysis of 417 individuals with XLAS in one series, which revealed that six individuals (1.4%, 95% confidence interval 0.6–3%) carried an additional *COL4A3* or *COL4A4* likely pathogenic variant [26].

## Q4: WHEN TO ORDER ALPORT GENE TESTING? WHAT IS THE LIST OF CLINICAL AND/OR HISTOLOGICAL FINDINGS THAT REQUIRE EXCLUDING ALPORT DIAGNOSIS IN DIFFERENTIAL DIAGNOSTICS AND WHAT SHOULD BE DONE IN MINORS?

**4.1-Recommendation: Indications for genetic testing for AS should include any of these:**

1. **Children and young adults (especially females of childbearing age) with isolated persistent glomerular (dysmorphic) haematuria**
2. **Individuals with persistent haematuria and family history of either well-documented haematuria or unexplained KF (at least in one first- or second-degree relative)**
3. **Kidney biopsy with characteristic findings (Box 1)**
4. **Individuals with persistent haematuria and high tone sensorineural hearing loss**
5. **Individuals with persistent haematuria and certain ocular findings (fleck retinopathy and anterior lenticonus).**

**When genetic testing is done to investigate proteinuric kidney disease, cystic kidney disease, unexplained KF, or hearing loss, *COL4A3/4/5* gene testing should be included (Box 2).** (Grade B, strong)

Genetic testing for AS should comprise analysis of all three *COL4A3/4/5* genes. Comprehensive analysis for single nucleotide variants and structural variants comprising entire coding sequences and adjacent intronic sequences (minimum ± 5) of these three genes should be performed. In addition, structural analysis should include the first four exons of *COL4A6* [6]. Awareness should be made that complex rearrangements, small copy-number variants and changes within regulatory fragments (promotor, introns, enhancers, silencers, etc.) might be missed by standard protocols that only target exons. The interpretation of variant pathogenicity should be performed in line with current state-of-art guidelines (e.g. American College of Medical Genetics/Association of Molecular Pathologists [ACMG/AMP] criteria, ACGS Best Practice Guidelines for Variant Classification, local recommendations) taking into account several disease-particularities (e.g. variant frequency thresholds and the position in the Gly-X-Y motifs) [1, 27, 28]. For autosomal recessive and digenic disease it is crucial to test the parents of the index case, if possible, to establish phase and help in assessing the pathogenicity of a detected variant.

The experts acknowledge that genetic screening strategies might vary depending on country-specific peculiarities including availability and access to genetic testing and reimbursement policy of national healthcare systems.

Box 1. **Histological findings that prompt genetic testing of *COL4A3/4/5* genes**

1. Basket weave-type lamellation of the GBM [29], ‘irregular bulging’ of the GBM [30]
2. Thinned GBM [29]
3. Electron-dense deposits in the GBM [31], different from immune complexes
4. Lucencies in the GBM [30]
5. Abnormal immunostaining of basement membranes for collagen IV-chains in expert labs [32]

Box 2. **Panels that should include *COL4A3/4/5* gene analysis**

1. Kidney panels:
  - Persistent haematuria
  - Proteinuric kidney disease
  - Focal segmental glomerulosclerosis (FSGS)
  - Podocytopathies
  - KF of unknown origin
2. Inner ear hearing loss

Note: *COL4A3* and *COL4A4* may be included in cystic kidney disease panels [33]

**4.2-Recommendation: The experts support the need for genetic testing in minors presenting signs of renal and/or extra-renal impairment belonging to Alport spectrum but refrain from proposing genetic testing in an asymptomatic child (including absence of haematuria), without the family having received genetic counselling.** (Grade B, strong)

In the case of minors, genetic counselling advocates advising parents to defer elective genetic testing until adulthood to prevent potential harms such as stigma, discrimination, and the loss of the child's ability to make her/his own decisions as an adult [34]. However, consensus on this policy may differ in the context of childhood-onset diseases like AS, where early management protocols are available. Regular clinical evaluation of haematuria (urinalysis) should be offered in the first place to all children born to patients with AS, irrespective of parental subtype (XL, AD, AR), except for boys with fathers with XLAS, who are not at risk (see Q12 for details).

## Q5: SHOULD GENETIC TESTING IN RELATIVES BE ENCOURAGED? SHOULD IT BE LIMITED TO (FAMILY) VARIANT TESTING OR RATHER COVER ALL THREE GENES? SHOULD PARTNERS BE TESTED WHEN WISHING TO CREATE A FAMILY?

**5.1-Suggestion: Where the molecular diagnosis of AS is established in an index case, genetic testing for that variant could be offered to all adult relatives at risk of inheriting the disease.** (Grade D, expert opinon)

A positive genetic testing should be accompanied by clinical evaluation of relatives at risk, including basic laboratory and image (ultrasound), kidney function assessment, and detailed pedigree analysis. Accordingly, the experts do not endorse consumer-initiated genetic testing services [34]. Testing of each second-degree relative should be preceded by an attempt to offer to test the intervening first-degree relative of the index case. Genetic testing in adult relatives should concern only the pathogenic variant(s) identified in the index case. It should be noted that approximately 10–15% of *COL4A5* and an unknown rate of *COL4A3/COL4A4* cases occur *de novo* [35].

In the case of a couple undergoing preconception counselling, a genetic test can be offered to the partner of the affected individual if detection of a high risk of ARAS would influence their decision-making or lead to reproductive intervention. In case of consanguinity, a test for the specific variant(s) present in the proband can be offered to the partner. In the absence of consanguinity, if the partner has persistent haematuria or a family history possibly fitting *COL4A3/4/5-*related kidney disease, full genetic testing of the three genes should be performed if this would influence reproductive decisions. The likelihood of having an offspring affected by ARAS for a patient with a monoallelic or biallelic P/LP variant and an unrelated, asymptomatic partner who does not have haematuria is less than 0.5%. As a result, genetic testing of the partner before starting a family is generally not recommended.

The timing of the genetic test, as well as decisions about preimplantation genetic diagnosis and prenatal genetic testing, should be discussed regarding local financial, social, and legal considerations.

## Q6: SHOULD ALPORT GENE TESTING BE INCLUDED IN GENE PANELS DESIGNED FOR KIDNEY DISEASES?

**6-Recommendation: Where genetic testing is done to investigate proteinuric kidney disease, unexplained KF or possibly cystic kidney disease, *COL4A3/4/5* gene testing should be included.** (Grade B, strong)

There are numerous reports of AS being diagnosed following genetic testing in patients with various kidney disease presentations, such as proteinuria, glomerulonephritis, focal segmental glomerulosclerosis, podocytopathies, IgA nephropathy, cysts, and unexplained KF. This suggests that it is logical to include AS genes in kidney disease gene panels. However, given the frequency of heterozygous P/LP variants in *COL4A3* and *COL4A4* in the general population, caution should be exercised before asserting that the identified variant fully explains the phenotype. This may require exclusion of an additional genetic or acquired kidney disease.

## Q7: SHOULD ALPORT GENE TESTING BE INCLUDED ON THE LIST OF SECONDARY FINDINGS/ACTIONABLE FINDINGS?

**7-Suggestion: Where genomic testing was performed for an indication other than to investigate kidney disease, a secondary finding of pathogenic variants in the *COL4A5* gene and cases with two or more pathogenic variants in *COL4A3/COL4A4* should be reported.** (Grade C, moderate)

Pathogenic variants in the *COL4A5* gene and cases where two or more P/LP variants in *COL4A3/COL4A4* are observed should be reported to the patient so that clinical evaluation of kidney function and co-segregation studies (to verify their biallelic character) might be undertaken.

More data on disease risk attributable to heterozygous P/LP autosomal variants in *COL4A3/COL4A4* is needed before these are reported to patients undergoing genomic testing for reasons unrelated to evidence of kidney disease.

## Q8: GIVEN THE RELATIVELY HIGH FREQUENCY OF INDIVIDUALS WITH HETEROZYGOUS *COL4A3/4* VARIANTS IN THE GENERAL POPULATION AND THEIR POTENTIALLY DETERIORATING EFFECT ON THE PHENOTYPE, SHOULD PATIENTS WITH PATHOGENIC *COL4A3/4* VARIANTS BE SCREENED FOR OTHER KIDNEY DISEASES IN THE CLINICAL PRACTICE?

**8-Suggestion: Caution should be exercised before asserting that a heterozygous P/LP *COL4A3/COL4A4* variant fully explains the phenotype. This may require exclusion of an additional genetic or acquired kidney diseases.** (Grade C, moderate)

The clinical impact of a heterozygous pathogenic variant in the *COL4A3/4/5* genes should be interpreted in the context of the clinical presentation of the tested individual. Accordingly, basic laboratory, imaging (ultrasound), kidney biopsy assessment and/or large panels of genes involved in (glomerular) kidney diseases should be considered before making the final genetic diagnosis.

## Q9: WHAT IS THE SENSITIVITY OF CURRENT GENETIC TESTS FOR AS? WHAT ARE THE POTENTIAL DIAGNOSTIC TOOLS IF NEXT-GENERATION SEQUENCING (NGS)-BASED GENETIC TESTING IS NEGATIVE?

**9-Statement: No targeted gene panel is 100% sensitive for the different types of genetic changes that can cause AS. In well-designed gene panels the sensitivity of *COL4A3/4/5* gene analysis to detect pathogenic variants is estimated to exceed 85%.** (Grade D, expert opinon)

The sensitivity of genetic testing has significantly improved in recent years; however, limitations remain in regions of the genome that are inadequately covered or unanalysed. In a Japanese study with comprehensive targeted sequencing using NGS (including search for structural variants) in which strong clinical inclusion criteria were used, the mutation detection rate was 86% [36]. The lower ratios reported in other cohorts [37] are due to less stringent inclusion criteria. Exome sequencing has lower sensitivity/detection rates [38].

When *COL4A3/4/5* gene testing and subsequent analysis of the larger panels of genes involved in (glomerular) kidney diseases are negative, the possibility of deep-intronic, regulatory, and structural variants such as inversions that might have been overlooked should be considered.

In such cases, having strong clinical suspicion of AS, targeted cDNA on skin RNA (for X-linked forms) or preferably urine RNA testing for the three genes should be considered; alternatively, or in parallel, genome sequencing might be attempted [39, 40].

## Q10: WHAT IS THE ESTIMATED LIFELONG RELATIVE RISK OF STAGE 5 CKD IN INDIVIDUALS WITH HETEROZYGOUS *COL4A3/COL4A4* VARIANTS VERSUS THAT IN THE GENERAL POPULATION?

**10.1-Statement: The risk of reaching KF at advanced age is higher for individuals with heterozygous P/LP *COL4A3/COL4A4* variants compared with the general population.** (Grade B, strong)

**10.2-Statement: Nephroprotective therapy can significantly reduce this risk.** (Grade B, strong)

It is crucial to provide the most accurate prognosis possible for the risk of stage 5 CKD, as KF poses the highest morbidity risk among individuals with AS. Heterozygous P/LP *COL4A3/COL4A4* variants, present in approximately 1% of the population [10] are associated with an expectedly low risk of CKD G5. The kidney prognosis of AS is significantly influenced by early treatment with renin-angiotensin system (RAS) inhibitors. However, studies based on hospital-based cohorts of patients and families with heterozygous P/LP *COL4A3/COL4A4* variants may suffer from ascertainment bias, skewing towards more severely affected individuals. Additionally, calculating the risk in affected families without excluding the index patient may also lead to overestimation.

Recent studies examining the frequency of P/LP *COL4A3/COL4A4* and *COL4A5* variants in hospital-based cohorts with KF suggest that heterozygous P/LP *COL4A3* and *COL4A4* variants occur about as frequently as P/LP *COL4A5* variants [14, 41–47]. Gibson *et al*. found that P/LP *COL4A3* and *COL4A4* variants were about 20 times more common than P/LP *COL4A5* variants in population datasets of participants without known kidney genetic disease [10]. Consequently, individuals with heterozygous P/LP *COL4A3*/*COL4A4* variants have a much lower risk of CKD progression than those with *COL4A5* pathogenic variants. Yet it is important to acknowledge that the risk associated with heterozygosity for a *COL4A3*/*COL4A4* variant in a family known to be affected by severe kidney disease is likely higher than in unselected populations, given the shared genetic and environmental background. Consequently, these are the families that often seek and receive genetic advice.

Based on population studies, the risk of CKD G5 by the age of 60 years in individuals with heterozygous P/LP *COL4A3/COL4A4* variants is <3%, and just above 3% by the age of 80 [18]. However, this risk may be modified by nephroprotective therapies [48]. Risk factors for estimated glomerular filtration rate (eGFR) decline are thought to be similar to those for other kidney diseases, and include hypertension, cardiovascular disease, diabetes, and obesity. Polygenic risk scores may play a certain role to explain phenotypic heterogeneity in AS, but further studies are needed [48]. Albuminuria (urine albumin : creatinine ratio; UACR) remains the best marker to evaluate the risk of eGFR decline. A summary of prognostic and progression factors is presented in Table 1 (Q15).

## Q11: WHAT IS THE ESTIMATED LIFELONG RELATIVE RISK AND AGE-RELATED RISK OF STAGE 5 CKD DEPENDING ON PATTERN OF INHERITANCE AND SEX?

**11.1-Statement: The majority of untreated XLAS males will reach KF in the second to fifth decade of life.** (Grade A, strong)

**11.2-Statement: The vast majority of XLAS males progress to CKD G5 with a variable speed of progression influenced by genotype, timing of nephroprotective therapy, and other environmental factors.** (Grade B, strong)

**11.3-Statement: XLAS females have a variable kidney outcome.** (Grade A, strong)

**11.4-Statement: This is probably mostly influenced by variable chromosome X inactivation, early start of nephroprotective therapy, and other environmental factors.** (Grade C, moderate)

**11.5-Statement: There is currently no evidence that there are sex differences in clinical symptoms and incidence for individuals with heterozygous P/LP *COL4A3* or *COL4A4* variants and individuals with ARAS.** (Grade B, strong)

**11.6-Statement: ARAS patients all progress to KF with a variable speed of progression influenced by genotype, early start of nephroprotective therapy, and other environmental factors. The majority of untreated ARAS patients will require kidney replacement therapy (KRT) in the second to third decade of life.** (Grade B, strong)

### XLAS—Male

Large cohorts of untreated XLAS males differ regarding the age at reaching KF. According to Jais *et al*., the median age at developing KF overall was 25 years; 90% of untreated patients had reached KF by the age of 40 [49]. In the study of Bekheirnia *et al*., the mean age at initiation of KRT was 37 years [50]. Genotype-phenotype correlation exists, with truncating variants causing a more severe kidney disease and earlier need for KRT in males [49–51]. Less severe pathogenic variants have been reported, such as p.Gly624Asp associated with later onset of KF [52–54]. The European data registry indicates that age at which KF occurs has gradually increased by more than five years since around 2005. This likely results from the increasing use of RAS blockade but could also be influenced by the increase of genetic testing in cases with milder disease, and the detection of milder variants [55].

### XLAS—Female

Overall, 20% of untreated women with a *COL4A5* variant have KF by 60 years. However, this risk is significantly reduced to less than 10% with adequate nephroprotective strategies [56]. There is no clear genotype-phenotype correlation in females.

### ARAS—both genders

Untreated individuals affected by ARAS typically reach KF in the second to third decade of life [25, 57]. There are no sex differences in clinical symptoms and incidence. Genotype-phenotype correlation studies suggest that the presence of two truncating variants of *COL4A3* or *COL4A4* is associated with earlier progression to KF and that the presence of two missense variants is generally associated with later onset of KF [25, 58].

### Individuals with a heterozygous P/LP *COL4A3/COL4A4* variant

The risk of reaching KF in this population is much lower than for XLAS and ARAS. There does not appear to be a sex difference in clinical symptoms or incidence. However, as this condition mostly involves elderly people and CKD is usually more severe in males, in large cohorts, a sex effect may be detected in the future.

Caveats to these estimates are the limited number of large unbiased published cohorts, the coexistence of other kidney conditions as these individuals may be elderly and have comorbidities, the extremely variable penetrance, and the possibility that these variants are not fully disease-causing but rather risk factors for the development of CKD.

## Q12: WHAT IS THE SUGGESTED FOLLOW-UP FOR INDIVIDUALS WITH HETEROZYGOUS P/LP *COL4A3/COL4A4* VARIANTS?

**12.1-Suggestion: In asymptomatic adults with heterozygous P/LP *COL4A3/COL4A4* variants, we suggest performing regular screenings (1- to 2-year intervals) for microhaematuria and microalbuminuria (employing UACR), along with annual blood pressure monitoring. The interval of screening should be individualized based on age, familial history, and comorbidities.** (Grade D, expert opinion)

**12.2-Suggestion: In individuals with microalbuminuria (defined by UACR > 30 mg/g on two distinct urine samples) and after initiation of renin-angiotensin-aldosterone system (RAS) blockers, we suggest monitoring eGFR and microalbuminuria every 6 months to 1 year. In individuals with CKD G2–G5, the interval for eGFR and UACR/UPCR monitoring should be adjusted to CKD stage and treatments.** (Grade D, expert opinion)

**12.3-Suggestion: In at-risk children and in children diagnosed with heterozygous P/LP *COL4A3/COL4A4* variants (for instance diagnosed in the setting of persistent haematuria), we suggest monitoring urinalysis every 1–4 years from 4–6 years old.** (Grade D, expert opinion)

There is a large variability in terms of disease severity in individuals with heterozygous P/LP *COL3A4/COL4A4* variants, ranging from asymptomatic to presentation with haematuria alone or with proteinuria, hypertension, and possible kidney function decline with subsequent KF in a subset of individuals [17]. Individuals with heterozygous P/LP *COL4A3/COL4A4* variants very rarely have the hearing loss or ocular abnormalities, typical of XLAS or ARAS [16–18, 59].

In a large cohort of 252 individuals with heterozygous pathogenic variants of *COL4A3* or *COL4A4* from 82 families, tested due to a positive family history of microhaematuria and proteinuria, or abnormal GBM in the proband, microhaematuria, and proteinuria were present in 92% and 65% of cases, respectively. Additionally, 24.6% reached KF at the median age of 67 years [17]. A systematic review of 777 affected individuals from 258 families in 48 publications yielded similar results with, amongst those with available data, 94.8% microhaematuria, 46.4% proteinuria, and KF in 15% [16]. However, because the aforementioned studies include individuals evaluated in hospitals, they are likely to be biased towards more severe disease [18]. Indeed, publicly available databases indicate that predicted pathogenic heterozygous variants of *COL4A3* and *COL4A4* are present in 1 of 106 ‘ostensibly healthy’ individuals [10]. Overall, the risk of CKD G5 by the age of 60 years in individuals with heterozygous *COL4A3/COL4A4* variants is estimated to be <3%, and slightly >3% by the age of 80 [18].

## Q13: TO WHOM, WHEN, AND HOW SHOULD HEARING BE ASSESSED?

**13.1-Suggestion: In patients with ARAS and males with XLAS, we suggest a first hearing evaluation at 4 years of age, followed by a hearing evaluation every year until adulthood. In adulthood, we suggest a hearing evaluation every 3 years. We recommend hearing screening until the age of 50 and testing based on symptoms afterwards.** (Grade D, expert opinion)

**13.2-Suggestion: We do not recommend regular hearing evaluation in individuals with a heterozygous P/LP *COL4A3/COL4A4* variant and only suggest testing in patients with subjective hearing loss.** (Grade D, expert opinion)

**13.3-Suggestion: In female patients with XLAS we suggest hearing evaluation at diagnosis or when reaching adulthood and then every 5 years in the absence of hearing loss symptoms.** (Grade D, expert opinion)

**13.4-Suggestion: We suggest pure tone audiometry as a standard hearing test.** (Grade D, expert opinion)

Every patient with AS exhibits an elevated risk to develop hearing loss, albeit with a different likelihood justifying a stratification strategy. Male patients with XLAS and individuals with ARAS are at high risk of develop hearing loss, usually in adolescence but sometimes in childhood [57, 60]. Especially in childhood, close monitoring is advised as untreated hearing loss can lead to delayed speech and language development. In individuals with a heterozygous P/LP *COL4A3/COL4A4* variant and female patients with XLAS the occurrence of hearing loss is very rare [61].

We recommend pure tone audiometry in all patients. If hearing loss is present, additional speech audiometry quantifying speech intelligibility could be considered. In very young patients with unreliable pure tone audiometric thresholds, transient evoked otoacoustic emissions (TEOAE) can be considered if the middle ear status is normal, as well as auditory brainstem potentials (ABR).

## Q14: TO WHOM, WHEN AND HOW SHOULD OPHTHALMOLOGIC EXAM BE PERFORMED?

**14-Suggestion: A full ophthalmologic examination should be performed at the time of diagnosis, including dilated fundoscopy. Periodical eye examinations could be performed from the time of diagnosis for XLAS and ARAS.** (Grade D, expert opinion)

Each examination should include clinical examination of all ocular segments with a specific focus on cornea, lens, and retinal alterations [62, 63].

Retinal alterations, including dot-and-fleck retinopathy, temporal retinal thinning, staircase foveopathy, and hypoplastic foveal pit, as well as further retinal alterations should be documented using optical coherence tomography (OCT) if the age of the patients allows [64–66]. For children aged ≤12 years: Ensure compliance with national screening for refractive errors. In case of high refractive errors detected, an ophthalmologic referral and screening for lenticonus should be performed.

Cataract surgery in patients with AS holds specific challenges, leading to the recommendation of performance in specialized centres with experience regarding the unique situation of intraocular lens (IOL) calculation in this situation (i.e. disregarding anterior chamber depth in case of lenticonus).

In individuals with a heterozygous P/LP *COL4A3/COL4A4* variant and female patients with XLAS the occurrence of eye involvement is very rare [61].

## Q15: WHAT ARE THE PROGRESSION FACTORS IN AS?

Progression factors are provided in the following table 1.

**Table 1:** Progression factirs in Alport Syndrome.

	XLAS male	XLAS female	ARAS	*COL4A3/COL4A4* heterozygotes
Risk of KF	∼80% by 40 years	∼20% by 60 years	∼50% by 30 years	∼3% by 80 years
Extrarenal features	+++	+/++	+++	–
Allelic effect/Genotype phenotype correlations	Truncating variants are associated with earlier progression to ESKD [50].	No clear correlation between *COL4A5* variant location, type, and phenotype [51]. Some studies suggest that truncating variants are associated with a higher risk for developing proteinuria [67].	Presence of two truncating variants of *COL4A3* or *COL4A4* is associated with earlier progression to ESKD [25]. Presence of two missense variants is generally associated with later onset of KF [58]. Presence of one missense variant is associated with an intermediate severity [25].	Gly substitutions with highly destabilizing residue (Arg Glu Asp Val Trp) are associated with increased risk of haematuria [25, 28]. Substitutions adjacent to a non-collagenous interruption or amino or carboxy terminus were associated less often with haematuria [28]. Protein truncating variants of *COL4A3* less severe than non-truncating variants of *COL4A3* [59].
Examples of less severe alleles	p.Gly624Asp (however, families with more severe disease course also reported [52–54].)		*COL4A3* p.Leu1474Pro	
Clinical features associated with progression to ESKD	Early onset overt proteinuria. Lenticonus, central fleck retinopathy or temporal retinal atrophy more frequent in severe XLAS.	Detection of proteinuria before the age of 15 is associated with a higher risk of progression to ESKD [67]. Hearing loss is associated with higher risk of progression to ESKD. GBM thickening or lamellation.	Early onset overt proteinuria associated with earlier progression to ESKD.	Appearance of microalbuminuria is associated with an increased risk of subsequent renal function decline. Histology: FSGS, GBM thickening and lamellation. Evidence of progression in affected relatives.

ARAS, autosomal recessive Alport syndrome; ESKD, end-stage kidney disease; FSGS, focal segmental glomerulosclerosis; GBM, glomerular basement membrane; KF, kidney failure; XLAS, X-linked Alport syndrome.

## Q16: WHAT IS THE ROLE OF KIDNEY BIOPSY IN PATIENTS WHO ALREADY HAVE A GENETIC DIAGNOSIS OF (OR FAMILY HISTORY OF CONFIRMED) AS?

**16.1-Suggestion: A kidney biopsy is not needed in individuals with a genetic diagnosis of XLAS or ARAS.** (Grade D, expert opinion)

**16.2-Suggestion: A kidney biopsy can sometimes be useful in individuals with heterozygous P/LP *COL4A3/COL4A4* variants because other aetiologies of glomerular diseases may coexist.** (Grade D, expert opinion)

**16.3-Suggestion: In individuals with heterozygous P/LP *COL4A3/COL4A4* variants and no microalbuminuria, over 40 years of age, who have been informed about the risk of subsequent development of CKD and want to donate a kidney, a kidney biopsy with electron microscopy should be discussed to evaluate the risk of developing severe CKD post-donation.** (Grade D, expert opinion)

Due to the relatively common occurrence of P/LP variants in the *COL4A3/COL4A4* genes in the general population, the experts emphasize the importance of considering kidney biopsy, especially in the absence of a family history [10]. This approach is critical for avoiding the oversight of potential diagnoses of kidney disease that may require different therapeutic interventions. For individuals with kidney disease and heterozygous P/LP variants in the *COL4A3/A4* genes, it is advisable to pursue familial co-segregation analysis. This will help to confirm that the identified variant is indeed causally contributing to the observed kidney phenotype. In singletons with heterozygous P/LP *COL4A3/A4* variants, the experts assume that genetic testing alone is not sufficient to obtain a diagnosis of precision, and that kidney biopsy might be needed. In addition, the experts emphasize that in case of unexpected disease course in individuals with heterozygous P/LP *COL4A3/A4* variants (e.g. rapid degradation of kidney function, unexplained acute kidney injury, sudden onset of nephrotic-range proteinuria), a kidney biopsy should be discussed to exclude superimposed kidney disease.

## Q17: WHEN (CONSIDERING BOTH AGE AND CLINICAL SITUATION) SHOULD RAS BLOCKERS BE STARTED IN A PATIENT WITH A *COL4A3/4/5* VARIANT?

**17.1-Recommendation: Early initiation of RAS blockade treatment is recommended in AS, with the most extensive evidence for ramipril due to the most abundant preclinical and clinical studies on safety and efficacy.** (Grade B, strong)

**17.2-Recommendation: RAS blockers should be initiated and titrated to maximum dose when microalbuminuria is detected irrespective of the genetic subgroup.** (Grade B, strong)

**17.3-Suggestion: In males with XLAS and in both sexes with ARAS, after 2 years of age, we suggest initiating RAS blockers as soon as microscopic haematuria is detected.** (Grade C, moderate)

**17.4-Suggestion: In heterozygous individuals with autosomal AS or females with heterozygous XLAS, we suggest not to start RAS blockers if they only show isolated microscopic haematuria.** (Grade D, expert opinion)

Early RAS blockade is the standard of care therapy in AS and it is supported by consistent preclinical and clinical data [5, 68–70].

A recent meta-analysis suggested that [71]:

- RAS blockers could be considered as a specific therapy for patients with AS to delay KF with any genetic type, especially at the early stage of the disease, and
- Additional therapy should be given in combination with RAS blockade as standard of care.

The classification of RAS blockade as a specific therapy for AS is a consequence of this meta-analysis and is based on the pathogenesis of AS. In brief, the type IV collagen defect leads to abnormal GBM assembly and altered turnover of GBM components. This abnormal GBM is believed to be vulnerable to mechanical load, leading to podocyte loss once several nephrons are damaged, and to FSGS in the remaining nephrons. Later in the course of the disease hypertension, the release of profibrotic proinflammatory chemokines, tubulointerstitial inflammation, and fibrosis may occur [72–74]. RAS blockade may influence various points of this pathogenesis without all mechanisms being exactly known.

### Efficacy and dosing of RAS blockade

Ramipril, an angiotensin-converting enzyme (ACE)-inhibitor with a target dose of 6 mg/m², is the most extensively studied drug in AS, both preclinically and clinically. It has been evaluated more thoroughly than other ACE-inhibitors and angiotensin receptor blockers (ARBs), including a randomized controlled trial (RCT) involving children with AS aged 2 years and older. Tables 2 (ACE-inhibitors) and 3 (ARBs) show the dosing and maximum doses of RAS blockade in children.

**Table 2:** First-line therapy in children (ACE-inhibitors).^a^

Agent	Dose
Ramipril	Start 1 to 2 mg/m^2^/day; max. 6 mg/m^2^/day
Enalapril	
Lisinopril	
Benazepril	
Fosinopril	
Quinapril	
Cilazapril	
Perinopril	
Trandolapril	

Modified from [70]

a The use of ACE-inhibitors is off-label use in non-hypertensive children with CKD or proteinuria. Commonly used off-label dosages in children can differ from country to country, therefore no dose recommendations for children other than for ramipril can be made [75].

**Table 3:** Second-line therapy in children (ARBs).^a^

Agent	Dose
Losartan	12.5 mg/m^2^/day; max. 50 mg/m^2^/day
Candesartan	
Irbesartan	
Telmisartan	
Valsartan	
Epresartan	

Modified from: [70]

a The use of ARBs is off-label use in non-hypertensive children with CKD or proteinuria. Commonly used off-label dosages in children can differ from country to country, therefore no dose recommendations for children other than for ramipril can be made [75].

Ramipril was superior to the AT1-antagonist candesartan in an Alport mouse model, in terms of preserving kidney function, antifibrotic and anti-inflammatory effects, positive effects on intracellular type IV collagen trafficking, and cell-protective properties via the local angiotensin system in podocytes. These findings were regardless of the blood pressure lowering effect and showed a strong correlation between early (pre-emptive) onset of ramipril therapy and the extent of nephroprotection with a more than doubling of lifespan until KF [76–78]. Recent data from another preclinical RCT confirm this strong correlation between early initiation of ramipril and maximum nephroprotection and suggest a role of mineralocorticoid receptor antagonists (MRA) [79].

Timely RAS blockade delays KF and improves life-expectancy in both ARAS and XLAS [80]. RAS blockade can delay KF by a median time of 18 years, if therapy is initiated with still preserved kidney function before nephron-loss, hyperfiltration, and hypertension speed up the disease [72, 80]. Safety, tolerability, and efficacy of ramipril have been studied in a placebo-controlled, double-blinded RCT in children with early stages of AS at 2 years of age and older with an up to 6-year treatment phase [75].

The genotype in AS influences the response to RAS blockade. Patients with more severe variants (such as truncating) have a more vulnerable GBM and respond less than patients with missense-variants [57, 81]. Timely RAS blockade can delay KF or even hold disease progression in heterozygous females with XLAS and heterozygous autosomal patients with albuminuria [56, 82].

Response to RAS blockade can be best measured by decrease of UACR, with high UACR being a predictor of faster disease progression [75, 83]. The therapeutic effect of RAS blockade will decrease over time due to various reasons such as increased aldosterone-levels (also known as aldosterone escape) [78], which provides the rationale for MRA-antagonists [79].

### Safety of RAS-blockade and off-label use in children

Neither ACE-inhibitors nor AT1-antagonists are approved for use in children with CKD, so their use in children with AS is off-label.

Safety of ramipril in children with CKD has been investigated in two phase III multi-year clinical trials, the ESCAPE trial in children with CKD and the EARLY PRO-TECT Alport trial in children 2 to 17 years of age with AS [75, 84]. In the placebo-controlled, double-blinded EARLY PRO-TECT Alport trial, ramipril could be up-titrated from 1 mg/m² to the maximum dosage of 6 mg/m² and without hypotensive side effects, ramipril showed a safety profile similar to placebo.

### Research objective RAS blockade in AS

Preclinical studies showed a stronger nephronprotective effect of the ACE-inhibitor ramipril versus the AT1-antagonist candesartan in the Alport animal model [76–78]. The experts recommend as an international research objective that observational data should be collected on the nephroprotective effects of ACE-inhibitors, AT1-antagonists, and MRA-antagonists in AS.

## Q18: SHOULD SGLT2-INHIBITORS BE USED IN AS? IF SO, WHEN SHOULD THEY BE PRESCRIBED?

**18.1-Statement: Existing evidence supports the use of SGLT2-inhibitors (SGLT2i) in adults with proteinuria and CKD and this benefit is likely to apply to individuals with AS.** (Grade D, expert opinion)

**18.2-Suggestion: In adults with AS and CKD G2A3 or higher (G3A1–3 and G4A1–3) with albuminuria, despite RAS blockers, the addition of a SGLT2i should be considered.** (Grade D, expert opinion)

**18.3-Statement: SGLT2i have not been trialled in children with CKD (no data available) and therefore a recommendation cannot be made on their use in children.** (Grade D, expert opinion)

**18.4-Suggestion: We suggest not to use SGLT2i as monotherapy without underlying RAS blockade.** (Grade C, moderate)

Dapagliflozin and empagliflozin are approved in the EU for the treatment of CKD (which includes AS) in adults (and post-pubertal teenagers in the UK), but not for children with CKD, as neither drug has been tested in this population. Dapagliflozin and empagliflozin have a marketing authorization for the treatment of Type 2 diabetes mellitus (T2DM) in children 10 years and older [85–87].

SGLT2i are approved for adults with CKD, including glomerular diseases such as AS, without eGFR-limitations in most countries or with the limitation of eGFR < 75–90 and >30 ml/min/1.73 m² in some countries. Existing evidence supports the use of SGLT2i in adults with proteinuria and CKD, and this benefit is likely to apply to individuals with AS [88].

Clinical trials have demonstrated positive cardiovascular and kidney outcomes of SGLT2i in adult patients with diabetic and other forms of CKD. Whether benefits extend to children, teenagers, and young adults with early-stage CKD is unknown as participants in the DAPA-CKD and EMPA-Kidney trials had a mean age of 60–65 years and CKD stages 3–4. The trials included less than 0.3% patients younger than 40 years of age with an eGFR >60 ml/min/1.73 m² [89, 90].

The DOUBLE PRO-TECT Alport trial (NCT05944016) with dapagliflozin will close this knowledge gap. The RCT started recruiting and aims to demonstrate superiority of the SGLT2i dapagliflozin in delaying change in UACR as a surrogate marker for CKD-progression in children and young adults at early stages of CKD in AS (age range 10 to 39 years; eGFR > 60 ml/min/1.73 m² in adults). A similar trial in children with any kind of CKD is being planned with empagliflozin.

### Efficacy of SGLT2i in AS

In preclinical studies empagliflozin reduces podocyte lipotoxicity in experimental AS [91], but did not show a significant delay of KF in monotherapy or on top of ramipril in mice with AS [79].

So far, no adult patients with AS with an eGFR > 60 ml/min/1.73 m² and no children have been investigated in RCTs with SGLT2i [89]. A small case series described a preliminary treatment response in adult patients with AS [92], and additional observational data on SGLT2i in AS have only been published in abstract form [93].

### Safety of SGLT2i and off-label use in children

SGLT2i show a good safety profile in adults with CKD. One case report in an adult patient with AS described a Fournier's gangrene possibly related to SGLT2i therapy [94]. The safety profile in children with CKD has not been investigated [95].

### Research objective SGLT2i in AS

The clinical research question related to SGLT2i is whether these drugs, by mechanistically acting on glomerular filtration pressure, are effective in children and adult patients with AS.

## Q19: SHOULD FURTHER STUDIES BE PERFORMED IN A PATIENT PRESENTING AT DIAGNOSIS WITH NEPHROTIC SYNDROME IN WHOM A *COL4A3/4/5* PATHOGENIC VARIANT IS IDENTIFIED?

**19.1-Suggestion: A kidney biopsy is suggested in an individual with a P/LP *COL4A3/4/5* variant if the onset of nephrotic syndrome is abrupt or the degree of proteinuria does not match the expected clinical presentation of the disease.** (Grade D, expert opinion)

**19.2-Suggestion: In the presence of an unexpected nephrotic syndrome and a non-specific kidney biopsy, we suggest expanding genetic testing by using a genetic panel test that includes all genes associated with inherited kidney disease or exome sequencing.** (Grade D, expert opinion)

The typical histological feature of later stages of AS is FSGS, which is thought to be triggered by progressive podocyte loss and consequent focal segmental glomerular scarring [72]. Although the exact patho-mechanisms are not well understood, most male patients with XLAS and males and females with ARAS with progressive disease can develop nephrotic range proteinuria and some even develop nephrotic syndrome [57, 68]. In heterozygous autosomal patients with AS, however, nephrotic syndrome is very rare and typically associated with additional contributing factors [56].

The amount of proteinuria can be aggravated by infections, poor blood pressure control, high body mass index, high protein intake, additional kidney disease, and during pregnancy [72].

In AS, nephrotic syndrome develops gradually over months and years, rather than presenting with an abrupt onset.

## Q20: COULD INDIVIDUALS WITH HETEROZYGOUS *COL4A3/COL4A4/COL4A5* PATHOGENIC VARIANTS BE CONSIDERED AS LIVING DONORS?

**20.1-Recommendation: We recommend determining the exact genotype in all possible donors.** (Grade B, strong)

**20.2-Suggestion: All relatives with heterozygous AS variants (males or females with heterozygous P/LP variants in *COL4A3*/*COL4A4* or females with a variant in *COL4A5*) should only be considered as the last possible resource for living kidney donation.** (Grade D, expert opinion)

**20.3-Suggestion: Living kidney donation is not advisable in individuals heterozygous for a single P/LP variant in *COL4A3, COL4A4*, or *COL4A5* aged under 40 years, or at any age if there is clinical or histological evidence of kidney damage (e.g. albuminuria, reduced eGFR or interstitial fibrosis/tubular atrophy greater than what would be normal for their age).** (Grade D, expert opinion)

**20.4-Suggestion: Where kidney donation is considered by an individual heterozygous for a single P/LP variant in *COL4A3, COL4A4*, or *COL4A5* aged over 40 years, in the absence of albuminuria or reduced eGFR, a kidney biopsy could be performed to detect evidence of subclinical kidney damage (i.e. scarring greater than what would be normal for their age) that would preclude donation. A decision to proceed with donation should only be made after careful consideration of the risks and benefits for that individual/family.** (Grade D, expert opinion)

**20.5-Suggestion: Lifelong monitoring (with prompt nephroprotective RAS blockade therapy if microalbuminuria or hypertension develop) could be offered to individuals heterozygous for a single P/LP variant in *COL4A3* or *COL4A4* or *COL4A5* who have donated a kidney, as for all living kidney donors.** (Grade D, expert opinion)

The experts are aware that when a young patient with AS needs KRT, the pressure on families to find a suitable living donor can be immense [96]. Additionally, waiting times for kidney transplants other than living donations vary by country—in Europe from 2–3 years to 8–10 years—leading to differing approaches in evaluating potential living donors with heterozygous variants of *COL4A3/COL4A4*. The high prevalence of *COL4A3* and *COL4A4* variants in the population also increases the likelihood that a donor will have one of these variants.

## Q21: WHAT IS THE PREVALENCE AND THE MANAGEMENT OF ALPORT POST-TRANSPLANT NEPHRITIS OR *DE NOVO* GBM DISEASE?

**21-Statement: While the risk of anti-GBM disease following a kidney transplant for AS without immunosuppressive therapy may be significant, current immunosuppressive treatments reduce this risk to less than 3%.** (Grade D, expert opinion)

AS is a genetic disease, which does not affect the transplanted kidney and does not re-occur in the transplanted kidney. Therefore, the long-term outcome after transplantation is better than for age-matched control patients with other kidney diseases [55].

The risk of anti-GBM disease in AS patients after transplantation is explained by the presence of alpha 3/4/5 type IV network in the transplanted ‘non-Alport’ kidney, which is unknown to the immune system. Risk of anti-GBM disease is higher in non-missense variants, which may lead to the absence of alpha 3/4/5 type IV collagen network in this individual, rather than in missense variants. A negative serological test does not exclude anti-GBM antibodies [97].

In some European countries, the occurrence of anti-GBM antibodies is monitored in patients with XLAS and ARAS, especially in truncating variants, in the first year (first 2 years) post-transplantation. The Goodpasture antigen is located in the NC1 domain of the alpha 3(IV) chain. Therefore, serological assays that specifically detect antibodies against this antigen, may yield negative results even in cases of anti-GBM disease. This can occur in situations where there is *de novo* exposure to the alpha 5(IV) chain, such as in XLAS recipients who develop an alloimmune response to the newly introduced protein [97, 98]. When anti-GBM antibodies or linear immune staining in the GBM/crescents are detected, augmenting/adding immunosuppression could be considered in the presence of kidney function deterioration.

## PATIENT NEEDS FOR PEOPLE WITH AS

Patient and family well-being is at the heart of this guideline for AS, a life-long genetic condition that can impact generations in a family. Depending on the severity of the genetic variant, different individuals or families can have clinical signs and symptoms on a spectrum from mild to severe. Even within a family, these can be different, and the differences cause a range of different patient journeys and needs. Patients should first understand where their genetic variant falls on the spectrum of potential outcomes for hearing, kidneys, and eyes. Prognosis information may be unavailable for their particular genetic variant, but patients need to know what is available and what research is going on to understand it better. Being diagnosed with a rare disease, like AS, can feel isolating Box 3 and Box 4.

Box 3. **What patients need at the point of their diagnosis**

1. Up-to-date clear and simple information about the outcomes for their genetic variant, if known. For children, information about the condition should be developmentally appropriate. Parents of affected children need reassurance that addressing the condition is crucial for helping their child have as normal a childhood as possible
2. Clinicians to connect them to their national patient organization, in order to reduce the feeling of isolation and to get peer support (see Supplementary Material for contact details of current European patient organizations).
3. Clarity on management and treatment
  - Current treatment options and research on future options
  - Latest lifestyle advice to follow (e.g. hydration, diet, cardiovascular exercise, no smoking, avoid nonsteroidal anti-inflammatory drugs [NSAIDs], etc.)
4. Genetic counselling to understand the impact of their genetic variant and the inheritance pattern. Given the complexity of genetics, patients need clear explanations to ensure that other family members are also offered genetic testing.
5. Offer of a follow-up conversation. After the shock of an unexpected diagnosis, patients often struggle to absorb all the necessary information. Therefore, a follow-up appointment soon after is important for addressing questions and gaining a deeper understanding.
6. Agreement on preferred methods and frequency for communication between appointments and how to contact the clinician with a query
  - What to do if the patient has a febrile episode and the blood in the urine increases?
  - How to manage medication, with diarrhoea and vomiting, etc.?

Box 4. **What patients need with ongoing management of their condition**

1. Encouragement to live life to the full. There are times along the journey when life can have a feeling of normality, and it is important to enjoy all those moments while you can.
2. To feel ‘in control’ as much as possible along their journey, despite outcomes sometimes being unknown. Feeling ‘in control’ reduces anxiety. Reducing anxiety also reduces the risk of depression. Many papers cite the psychosocial aspects of living with an inherited rare disease whether you are the patient or a parent, for example ‘parental guilt’ of passing on a gene. Patients may need psychological support.
3. To feel treated as an equal in the patient/clinician relationship, building an open and trusting partnership, with an understanding that you are on a long-term journey together. Patients need empowering to become advocates for their own care and the care of others.
4. A comprehensive assessment of the patient's ‘life goals’ and support to achieve them. For instance, if a young adult is starting work or college and is close to needing dialysis, decide together on the timing and method of dialysis to balance these goals with establishing treatment, or consider postponing the goals if necessary.
5. Support in school and working environment for children and young adults to help cope with hearing loss and wearing hearing aids as well as frequent absences for medical appointments.
6. Smooth transition to adult services as young adults leave school, moving onto work, college, university, moving away from the family home, and potentially starting relationships while managing declining hearing and declining kidney function. Young adult patients may also need family planning and/or contraceptive advice, and additional clinical support for women during pregnancy.
7. Regular assessment as to whether patients or family members need psychosocial support. On a patient journey, there can be critical crunch points, as the hearing starts to decline or kidney function declines:
  - Patients and patient families reaching dialysis and transplant need support adjusting to a different way of life.
  - Young, transplanted patients may require additional psychosocial post-transplant. Research indicates that up to 30% of transplanted patients lose their lives to poor mental health [99].
  - Extra support for siblings. These declining stages can make them feel ‘very out of control’ and cause mental health issues.
8. Financial support. There is a huge cost attending many medical appointments in terms of travel expenses and time away from work or school and medication costs. Research evidence shows that people living with rare kidney disease often face socio-economic challenges, with many unable to continue work [100].

Further information on support available for people living with AS is provided in the Supplementary Material.

## CLOSING REMARKS AND FUTURE PERSPECTIVES

This guideline provides clear directives on diagnosing and managing XLAS and ARAS. However, for individuals with heterozygous variants in *COL4A4* or *COL4A3*, the evidence is currently limited, and the recommendations may evolve as new information emerges. There is still a lack of evidence regarding the true prevalence and impact of these variants. In the near future, polygenic risk scores, specifically genome-wide polygenic scores, may play a significant role in interpreting these gene variants [48]. Larger, well-phenotyped population studies are needed to accurately assess the contribution of *COL4A4* or *COL4A3* pathogenic variants to CKD.

Besides that, the name of the disease is also undergoing an international renaming process.

Despite limited evidence for certain aspects of this guideline, the experts believe that addressing all facets of AS—a leading genetic cause of CKD and the second most common genetic cause of KF after autosomal dominant polycystic kidney disease—is crucial. This guidance, developed by a multidisciplinary team of internationally renowned experts and evaluated through a Delphi survey involving most of the remaining global experts, represents a significant contribution to the field.

## Supplementary Material

gfae265_Supplemental_File

## Data Availability

No new data were generated or analysed in support of this research.

## References

[1] ClinGen Alport Syndrome Variant Curation Expert Panel Recommendations. https://www.clinicalgenome.org/affiliation/50146/ (8 April 2024, date last accessed).

[2] Savige J, Rana K, Tonna S et al. Thin basement membrane nephropathy. Kidney Int 2003;64:1169–78. 10.1046/j.1523-1755.2003.00234.x 12969134

[3] Ouzzani M, Hammady H, Fedorowicz Z et al. Rayyan—a web and mobile app for systematic reviews. Syst Rev 2016;5:210. 10.1186/s13643-016-0384-4 27919275 PMC5139140

[4] Classifying recommendations for clinical practice guidelines. Pediatrics 2004;114:874–7. 10.1542/peds.2004-1260 15342869

[5] Savige J, Gregory M, Gross O et al. Expert guidelines for the management of Alport syndrome and thin basement membrane nephropathy. J Am Soc Nephrol 2013;24:364–75. 10.1681/ASN.2012020148 23349312

[6] Heidet L, Dahan K, Zhou J et al. Deletions of both alpha 5(IV) and alpha 6(IV) collagen genes in Alport syndrome and in Alport syndrome associated with smooth muscle tumours. Hum Mol Genet 1995;4:99–108. 10.1093/hmg/4.1.99 7711741

[7] Hasstedt SJ, Atkin CL. X-linked inheritance of Alport syndrome: family P revisited. Am J Hum Genet 1983;35:1241–51. 6650503 PMC1685969

[8] Persson U, Hertz JM, Wieslander J et al. Alport syndrome in southern Sweden. CN 2005;64:85–90. 10.5414/CNP64085 16114783

[9] Pajari H, Kääriäinen H, Muhonen T et al. Alport's syndrome in 78 patients: epidemiological and clinical study. Acta Paediatr 1996;85:1300–6. 10.1111/j.1651-2227.1996.tb13915.x 8955456

[10] Gibson J, Fieldhouse R, Chan MMY et al. Prevalence estimates of predicted pathogenic COL4A3-COL4A5 variants in a population sequencing database and their implications for Alport syndrome. JASN 2021;32:2273–90. 10.1681/ASN.2020071065 34400539 PMC8729840

[11] Robinson MR, Kleinman A, Graff M et al. Genetic evidence of assortative mating in humans. Nat Hum Behav 2017;1:0016. 10.1038/s41562-016-0016

[12] Karczewski KJ, Solomonson M, Chao KR et al. Systematic single-variant and gene-based association testing of thousands of phenotypes in 394,841 UK Biobank exomes. Cell Genomics 2022;2:100168. 10.1016/j.xgen.2022.100168 36778668 PMC9903662

[13] Gagliano Taliun SA, Sulem P, Sveinbjornsson G et al. GWAS of hematuria. CJASN 2022;17:672–83. 10.2215/CJN.13711021 35474271 PMC9269584

[14] Groopman EE, Marasa M, Cameron-Christie S et al. Diagnostic utility of exome sequencing for kidney disease. N Engl J Med 2019;380:142–51. 10.1056/NEJMoa1806891 30586318 PMC6510541

[15] Domingo-Gallego A, Pybus M, Bullich G et al. Clinical utility of genetic testing in early-onset kidney disease: seven genes are the main players. Nephrol Dial Transplant 2022;37:687–96. 10.1093/ndt/gfab019 33532864

[16] Matthaiou A, Poulli T, Deltas C. Prevalence of clinical, pathological and molecular features of glomerular basement membrane nephropathy caused by COL4A3 or COL4A4 mutations: a systematic review. Clin Kidney J 2020;13:1025–36. 10.1093/ckj/sfz176 33391746 PMC7769542

[17] Furlano M, Martínez V, Pybus M et al. Clinical and genetic features of autosomal dominant Alport syndrome: a cohort study. Am J Kidney Dis 2021;78:560–570.e1.e1. 10.1053/j.ajkd.2021.02.326 33838161

[18] Savige J. Heterozygous pathogenic COL4A3 and COL4A4 variants (autosomal dominant Alport syndrome) are common, and not typically associated with end-stage kidney failure, hearing loss, or ocular abnormalities. Kidney Int Rep 2022;7:1933–8. 10.1016/j.ekir.2022.06.001 36090501 PMC9458992

[19] Yuan X, Su Q, Wang H et al. Genetic variants of the COL4A3, COL4A4, and COL4A5 genes contribute to thinned glomerular basement membrane lesions in sporadic IgA nephropathy patients. JASN 2023;34:132–44. 10.1681/ASN.2021111447 36130833 PMC10101589

[20] Voskarides K, Damianou L, Neocleous V et al. COL4A3/COL4A4 mutations producing focal segmental glomerulosclerosis and renal failure in thin basement membrane nephropathy. J Am Soc Nephrol 2007;18:3004–16. 10.1681/ASN.2007040444 17942953

[21] Deltas C, Papagregoriou G, Louka SF et al. Genetic modifiers of Mendelian monogenic collagen IV nephropathies in humans and mice. Genes 2023;14:1686. 10.3390/genes14091686 37761826 PMC10530214

[22] Savige J. A further genetic cause of thin basement membrane nephropathy. Nephrol Dial Transplant 2016;31:1758–60. 10.1093/ndt/gfw217 27312149

[23] Savige J, Renieri A, Ars E et al. Digenic Alport syndrome. CJASN 2022;17:1697–706. 10.2215/CJN.03120322 35675912 PMC9718039

[24] Mencarelli MA, Heidet L, Storey H et al. Evidence of digenic inheritance in Alport syndrome. J Med Genet 2015;52:163–74. 10.1136/jmedgenet-2014-102822 25575550

[25] Storey H, Savige J, Sivakumar V et al. COL4A3/COL4A4 mutations and features in individuals with autosomal recessive Alport syndrome. J Am Soc Nephrol 2013;24:1945–54. 10.1681/ASN.2012100985 24052634 PMC3839543

[26] Zhang Y, Ding J, Zhang H et al. Effect of heterozygous pathogenic COL4A3 or COL4A4 variants on patients with X-linked Alport syndrome. Molec Gen & Gen Med 2019;7:e647. 10.1002/mgg3.647 PMC6503168 30883042

[27] Savige J, Storey H, Watson E et al. Consensus statement on standards and guidelines for the molecular diagnostics of Alport syndrome: refining the ACMG criteria. Eur J Hum Genet 2021;29:1186–97. 10.1038/s41431-021-00858-1 33854215 PMC8384871

[28] Gibson JT, Huang M, Shenelli C et al. Genotype-phenotype correlations for COL4A3-COL4A5 variants resulting in Gly substitutions in Alport syndrome. Sci Rep 2022;12:2722. 10.1038/s41598-022-06525-9 35177655 PMC8854626

[29] Spear GS, Slusser RJ. Alport's syndrome. Emphasizing electron microscopic studies of the glomerulus. Am J Pathol 1972;69:213–24. 4343992 PMC2032644

[30] Mazzucco G, Barsotti P, Muda AO et al. Ultrastructural and immunohistochemical findings in Alport's syndrome: a study of 108 patients from 97 Italian families with particular emphasis on COL4A5 gene mutation correlations. J Am Soc Nephrol 1998;9:1023–31. 10.1681/ASN.V961023 9621285

[31] Noël LH. Renal pathology and ultrastructural findings in Alport's syndrome. Ren Fail 2000;22:751–8. 10.1081/JDI-100101960 11104162

[32] Gubler MC, Knebelmann B, Beziau A et al. Autosomal recessive Alport syndrome: immunohistochemical study of type IV collagen chain distribution. Kidney Int 1995;47:1142–7. 10.1038/ki.1995.163 7783412

[33] Gulati A, Sevillano AM, Praga M et al. Collagen IV gene mutations in adults with bilateral renal cysts and CKD. Kidney Int Rep 2020;5:103–8. 10.1016/j.ekir.2019.09.004 31922066 PMC6943786

[34] Botkin JR, Belmont JW, Berg JS et al. Points to consider: ethical, legal, and psychosocial implications of genetic testing in children and adolescents. Am Hum Genet 2015;97:6–21. 10.1016/j.ajhg.2015.05.022 PMC4570999 26140447

[35] Heidet L, Gubler MC. The renal lesions of Alport syndrome. J Am Soc Nephrol 2009;20:1210–5. 10.1681/ASN.2008090984 19470679

[36] Yamamura T, Nozu K, Minamikawa S et al. Comparison between conventional and comprehensive sequencing approaches for genetic diagnosis of Alport syndrome. Molec Gen & Gen Med 2019;7:e883. 10.1002/mgg3.883 PMC6732293 31364286

[37] Morinière V, Dahan K, Hilbert P et al. Improving mutation screening in familial hematuric nephropathies through next generation sequencing. J Am Soc Nephrol 2014;25:2740–51. 10.1681/ASN.2013080912 24854265 PMC4243343

[38] Koboldt DC. Best practices for variant calling in clinical sequencing. Genome Med 2020;12:91. 10.1186/s13073-020-00791-w 33106175 PMC7586657

[39] Boisson M, Arrondel C, Cagnard N et al. A wave of deep intronic mutations in X-linked Alport syndrome. Kidney Int 2023;104:367–77. 10.1016/j.kint.2023.05.006 37230224

[40] Yamamura T, Horinouchi T, Aoto Y et al. The contribution of COL4A5 splicing variants to the pathogenesis of X-linked Alport syndrome. Front Med 2022;9:841391. 10.3389/fmed.2022.841391 PMC8861460 35211492

[41] Gillion V, Dahan K, Cosyns JP et al. Genotype and outcome after kidney transplantation in Alport syndrome. Kidney Int Rep 2018;3:652–60. 10.1016/j.ekir.2018.01.008 29854973 PMC5976824

[42] Ottlewski I, Münch J, Wagner T et al. Value of renal gene panel diagnostics in adults waiting for kidney transplantation due to undetermined end-stage renal disease. Kidney Int 2019;96:222–30. 10.1016/j.kint.2019.01.038 31027891

[43] Bullich G, Domingo-Gallego A, Vargas I et al. A kidney-disease gene panel allows a comprehensive genetic diagnosis of cystic and glomerular inherited kidney diseases. Kidney Int 2018;94:363–71. 10.1016/j.kint.2018.02.027 29801666

[44] Lata S, Marasa M, Li Y et al. Whole-exome sequencing in adults with chronic kidney disease: a pilot study. Ann Intern Med 2018;168:100–9. 10.7326/M17-1319 29204651 PMC5947852

[45] Mann N, Braun DA, Amann K et al. Whole-exome sequencing enables a precision medicine approach for kidney transplant recipients. JASN 2019;30:201–15. 10.1681/ASN.2018060575 30655312 PMC6362619

[46] Connaughton DM, Kennedy C, Shril S et al. Monogenic causes of chronic kidney disease in adults. Kidney Int 2019;95:914–28. 10.1016/j.kint.2018.10.031 30773290 PMC6431580

[47] Claus LR, Snoek R, Knoers N et al. Review of genetic testing in kidney disease patients: diagnostic yield of single nucleotide variants and copy number variations evaluated across and within kidney phenotype groups. Am J Med Genetics Pt C 2022;190:358–76. 10.1002/ajmg.c.31995 PMC9828643 36161467

[48] Khan A, Shang N, Nestor JG et al. Polygenic risk alters the penetrance of monogenic kidney disease. Nat Commun 2023;14:8318. 10.1038/s41467-023-43878-9 38097619 PMC10721887

[49] Jais JP, Knebelmann B, Giatras I et al. X-linked Alport syndrome: natural history in 195 families and genotype-phenotype correlations in males. J Am Soc Nephrol 2000;11:649–57. 10.1681/ASN.V114649 10752524

[50] Bekheirnia MR, Reed B, Gregory MC et al. Genotype-phenotype correlation in X-linked Alport syndrome. J Am Soc Nephrol 2010;21:876–83. 10.1681/ASN.2009070784 20378821 PMC2865738

[51] Jais JP, Knebelmann B, Giatras I et al. X-linked Alport syndrome: natural history and genotype-phenotype correlations in girls and women belonging to 195 families: a “European Community Alport Syndrome Concerted Action” study. J Am Soc Nephrol 2003;14:2603–10. 10.1097/01.ASN.0000090034.71205.74 14514738

[52] Macheroux EP, Braunisch MC, Pucci Pegler S et al. The hypomorphic variant p.(Gly624Asp) in COL4A5 as a possible cause for an unexpected severe phenotype in a family with X-linked Alport syndrome. Front Pediatr 2019;7:485. 10.3389/fped.2019.00485 31850286 PMC6887795

[53] Boeckhaus J, Hoefele J, Riedhammer KM et al. Lifelong effect of therapy in young patients with the COL4A5 Alport missense variant p.(Gly624Asp): a prospective cohort study. Nephrol Dial Transplant 2022;37:2496–504. 10.1093/ndt/gfac006 35022790

[54] Żurowska AM, Bielska O, Daca-Roszak P et al. Mild X-linked Alport syndrome due to the COL4A5 G624D variant originating in the Middle Ages is predominant in Central/East Europe and causes kidney failure in midlife. Kidney Int 2021;99:1451–8. 10.1016/j.kint.2020.10.040 33309955

[55] Temme J, Kramer A, Jager KJ et al. Outcomes of male patients with Alport syndrome undergoing renal replacement therapy. Clin J Am Soc Nephrol 2012;7:1969–76. 10.2215/CJN.02190312 22997344 PMC3513741

[56] Temme J, Peters F, Lange K et al. Incidence of renal failure and nephroprotection by RAAS inhibition in heterozygous carriers of X-chromosomal and autosomal recessive Alport mutations. Kidney Int 2012;81:779–83. 10.1038/ki.2011.452 22237748

[57] Zhang Y, Böckhaus J, Wang F et al. Genotype-phenotype correlations and nephroprotective effects of RAAS inhibition in patients with autosomal recessive Alport syndrome. Pediatr Nephrol 2021;36:2719–30. 10.1007/s00467-021-05040-9 33772369 PMC8370956

[58] Lee JM, Nozu K, Choi DE et al. Features of autosomal recessive Alport syndrome: a systematic review. J Clin Med 2019;8:178. 30717457 10.3390/jcm8020178 PMC6406612

[59] Solanki KV, Hu Y, Moore BS et al. The phenotypic spectrum of COL4A3 heterozygotes. Kidney Int Rep 2023;8:2088–99. 10.1016/j.ekir.2023.07.010 37849993 PMC10577321

[60] Zhang X, Zhang Y, Zhang Y et al. X-linked Alport syndrome: pathogenic variant features and further auditory genotype-phenotype correlations in males. Orphanet J Rare Dis 2018;13:229. 10.1186/s13023-018-0974-4 30577881 PMC6303895

[61] Rosado C, Bueno E, Fraile P et al. A new mutation in the COL4A3 gene responsible for autosomal dominant Alport syndrome, which only generates hearing loss in some carriers. Eur J Med Genet 2015;58:35–38. 10.1016/j.ejmg.2014.10.003 25450602

[62] Savige J, Sheth S, Leys A et al. Ocular features in Alport syndrome: pathogenesis and clinical significance. Clin J Am Soc Nephrol 2015;10:703–9. 10.2215/CJN.10581014 25649157 PMC4386265

[63] Colville DJ, Savige J. Alport syndrome. A review of the ocular manifestations. Ophthalmic Genet 1997;18:161–73. 10.3109/13816819709041431 9457747

[64] De Silva SR, Arno G, Robson AG et al. The X-linked retinopathies: physiological insights, pathogenic mechanisms, phenotypic features and novel therapies. Prog Retin Eye Res 2021;82:100898. 10.1016/j.preteyeres.2020.100898 32860923

[65] Hess K, Pfau M, Wintergerst MWM et al. Phenotypic spectrum of the foveal configuration and foveal avascular zone in patients with Alport syndrome. Invest Ophthalmol Vis Sci 2020;61:5. 10.1167/iovs.61.2.5 PMC7324255 32031577

[66] Ahmed F, Kamae KK, Jones DJ et al. Temporal macular thinning associated with X-linked Alport syndrome. JAMA Ophthalmol 2013;131:777–82. 10.1001/jamaophthalmol.2013.1452 23572034 PMC3746023

[67] Gibson JT, de Gooyer M, Huang M et al. A systematic review of pathogenic COL4A5 variants and proteinuria in women and girls with X-linked Alport syndrome. Kidney Int Rep 2022;7:2454–61. 10.1016/j.ekir.2022.08.021 36531881 PMC9751687

[68] Kashtan CE, Gross O. Clinical practice recommendations for the diagnosis and management of Alport syndrome in children, adolescents, and young adults—an update for 2020. Pediatr Nephrol 2021;36:711–9. 10.1007/s00467-020-04819-6 33159213

[69] Weinstock BA, Feldman DL, Fornoni A et al. Clinical trial recommendations for potential Alport syndrome therapies. Kidney Int 2020;97:1109–16. 10.1016/j.kint.2020.02.029 32386680 PMC7614298

[70] Kashtan CE, Ding J, Gregory M et al. Clinical practice recommendations for the treatment of Alport syndrome: a statement of the Alport Syndrome Research Collaborative. Pediatr Nephrol 2013;28:5–11. 10.1007/s00467-012-2138-4 22461141 PMC3505543

[71] Zeng M, Di H, Liang J et al. Effectiveness of renin-angiotensin-aldosterone system blockers in patients with Alport syndrome: a systematic review and meta-analysis. Nephrol Dial Transplant 2023;38:2485–93. 10.1093/ndt/gfad105 37218713

[72] Kruegel J, Rubel D, Gross O. Alport syndrome—insights from basic and clinical research. Nat Rev Nephrol 2013;9:170–8. 10.1038/nrneph.2012.259 23165304

[73] Funk SD, Lin MH, Miner JH. Alport syndrome and Pierson syndrome: diseases of the glomerular basement membrane. Matrix Biol 2018;71-72:250–61. 10.1016/j.matbio.2018.04.008 29673759 PMC6146048

[74] Naylor RW, Morais M, Lennon R. Complexities of the glomerular basement membrane. Nat Rev Nephrol 2021;17:112–27. 10.1038/s41581-020-0329-y 32839582

[75] Gross O, Tönshoff B, Weber LT et al. A multicenter, randomized, placebo-controlled, double-blind phase 3 trial with open-arm comparison indicates safety and efficacy of nephroprotective therapy with ramipril in children with Alport's syndrome. Kidney Int 2020;97:1275–86. 10.1016/j.kint.2019.12.015 32299679

[76] Gross O, Beirowski B, Koepke ML et al. Preemptive ramipril therapy delays renal failure and reduces renal fibrosis in COL4A3-knockout mice with Alport syndrome. Kidney Int 2003;63:438–46. 10.1046/j.1523-1755.2003.00779.x 12631109

[77] Gross O, Schulze-Lohoff E, Koepke ML et al. Antifibrotic, nephroprotective potential of ACE inhibitor vs AT1 antagonist in a murine model of renal fibrosis. Nephrol Dial Transplant 2004;19:1716–23. 10.1093/ndt/gfh219 15128880

[78] Rubel D, Zhang Y, Sowa N et al. Organoprotective effects of spironolactone on top of ramipril therapy in a mouse model for Alport syndrome. JCM 2021;2958 10: 10.3390/jcm10132958 34209341 PMC8268845

[79] Zhu Z, Rosenkranz KAT, Kusunoki Y et al. Finerenone added to RAS/SGLT2 blockade for CKD in Alport syndrome: results of a randomized controlled trial with Col4a3-/- mice. JASN 2023;34:1513–20. 10.1681/ASN.0000000000000186 37428955 PMC10482061

[80] Gross O, Licht C, Anders HJ et al. Early angiotensin-converting enzyme inhibition in Alport syndrome delays renal failure and improves life expectancy. Kidney Int 2012;81:494–501. 10.1038/ki.2011.407 22166847

[81] Yamamura T, Horinouchi T, Nagano C et al. Genotype-phenotype correlations influence the response to angiotensin-targeting drugs in Japanese patients with male X-linked Alport syndrome. Kidney Int 2020;98:1605–14. 10.1016/j.kint.2020.06.038 32712167

[82] Stock J, Kuenanz J, Glonke N et al. Prospective study on the potential of RAAS blockade to halt renal disease in Alport syndrome patients with heterozygous mutations. Pediatr Nephrol 2017;32:131–7. 10.1007/s00467-016-3452-z 27402170

[83] Boeckhaus J, Mohr L, Dihazi H et al. Ratio of urinary proteins to albumin excretion shifts substantially during progression of the podocytopathy Alport syndrome, and spot urine is a reliable method to detect these pathologic changes. Cells 2023;1333 12:. 10.3390/cells12091333 37174733 PMC10177071

[84] Wühl E, Trivelli A, Picca S et al. Strict blood-pressure control and progression of renal failure in children. N Engl J Med 2009;361:1639–50. 19846849 10.1056/NEJMoa0902066

[85] Tamborlane WV, Laffel LM, Shehadeh N et al. Efficacy and safety of dapagliflozin in children and young adults with type 2 diabetes: a prospective, multicentre, randomised, parallel group, phase 3 study. Lancet Diabetes Endocrinol 2022;10:341–50. 10.1016/S2213-8587(22)00052-3 35378069 PMC10851108

[86] European Medicines Agency (EMA). Forxiga (dapagliflozin). An overview of Forxiga and why it is authorised in the EU. 2023. https://www.ema.europa.eu/en/documents/overview/forxiga-epar-medicine-overview_en.pdf (8 April 2024, date last accessed).

[87] European Medicines Agency (EMA). Jardiance (empagliflozin): An overview of Jardiance and why it is authorised in the EU. 2023. https://www.ema.europa.eu/en/documents/overview/jardiance-epar-summary-public_en.pdf (8 April 2024, date last accessed).

[88] Mabillard H, Sayer JA. SGLT2 inhibitors—a potential treatment for Alport syndrome. Clin Sci (Lond) 2020;134:379–88. 10.1042/CS20191276 32064497

[89] Heerspink HJL, Stefánsson BV, Correa-Rotter R et al. Dapagliflozin in patients with chronic kidney disease. N Engl J Med 2020;383:1436–46. 10.1056/NEJMoa2024816 32970396

[90] Herrington WG, Staplin N, Wanner C et al. Empagliflozin in patients with chronic kidney disease. N Engl J Med 2023;388:117–27. 36331190 10.1056/NEJMoa2204233 PMC7614055

[91] Ge M, Molina J, Kim JJ et al. Empagliflozin reduces podocyte lipotoxicity in experimental Alport syndrome. eLife 2023;12:e83353. 10.7554/eLife.83353 37129368 PMC10185338

[92] Boeckhaus J, Gross O. Sodium-glucose cotransporter-2 inhibitors in patients with hereditary podocytopathies, Alport syndrome, and FSGS: a case series to better plan a large-scale study. Cells 2021;1815 10: 10.3390/cells10071815 34359984 PMC8303219

[93] Boeckhaus J, Gale D, Simon J et al. SGLT2-inhibition in patients with alport syndrome. Kidney Int Reports 2024;9:3490–3500. 10.1016/j.ekir.2024.09.014 PMC11652101 39698346

[94] Heidegger I, Zwierzina M, Boeckhaus J et al. Fournier's gangrene in a patient with CKD without diabetes possibly related to sodium-glucose cotransporter 2 inhibitor therapy. Kidney Int Rep 2024;9:1531–3. 10.1016/j.ekir.2024.02.1404 38707800 PMC11068935

[95] Gross O, Haffner D, Schaefer F et al. SGLT2 inhibitors: approved for adults and cats but not for children with CKD. Nephrol Dial Transplant 2024;39:907–9. 10.1093/ndt/gfae029 38308509

[96] Gross O, Weber M, Fries JW et al. Living donor kidney transplantation from relatives with mild urinary abnormalities in Alport syndrome: long-term risk, benefit and outcome. Nephrol Dial Transplant 2009;24:1626–30. 10.1093/ndt/gfn635 19028755

[97] Brainwood D, Kashtan C, Gubler MC et al. Targets of alloantibodies in Alport anti-glomerular basement membrane disease after renal transplantation. Kidney Int 1998;53:762–6. 10.1046/j.1523-1755.1998.00794.x 9507224

[98] Wang XP, Fogo AB, Colon S et al. Distinct epitopes for anti-glomerular basement membrane Alport alloantibodies and Goodpasture autoantibodies within the noncollagenous domain of alpha3(IV) collagen: a janus-faced antigen. J Am Soc Nephrol 2005;16:3563–71. 10.1681/ASN.2005060670 16236801

[99] Wilton J. Addressing the mental health challenges of life with kidney disease: The case for change. Centre for Mental Health & Kidney Research, Peterborough, UK; 2023. Available at: https://www.kidneyresearchuk.org/wp-content/uploads/2023/05/CentreforMHKRUK_TheCaseForChange.pdf (8 April 2024, date last accessed).

[100] Wong K, Pitcher D, Braddon F et al. Effects of rare kidney diseases on kidney failure: a longitudinal analysis of the UK National Registry of Rare Kidney Diseases (RaDaR) cohort. Lancet North Am Ed 2024;403:1279–89. 10.1016/S0140-6736(23)02843-X PMC11750427 38492578

