# Supplementary material for: Diagnosis, management and treatment of the Alport syndrome – 2024 guideline on behalf of ERKNet, ERA and ESPN
Source: Nephrol Dial Transplant. 2024 Dec 2;40(6):1091–106. doi: 10.1093/ndt/gfae265 (PMC12209846; doi:10.1093/ndt/gfae265)
Supplement: gfae265_Supplemental_File [file gfae265_Supplemental_File.docx]

**Diagnosis, management and treatment of the Alport syndrome – 2024 guideline on behalf of ERKNet, ERA and ESPN**

**Supplementary material**

***Appendix 1: Glossary***

**PERSISTENT HEMATURIA:** The presence of red blood cells in the urine (≥ 3 red blood cells per high-power field), confirmed by at least two out of three consecutive samples over a period of at least six months[1].

**ALBUMINURIA GRADING[1]:**

A1: Normal to mildly increased: ACR <30 mg/g (ACR <3 mg/mmol)

A2: Moderately increased (previously termed microalbuminuria): ACR 30-300 mg/g (ACR 3-30 mg/mmol)

A3: Severely increased: ACR >300 mg/g (ACR >30 mg/mmol)

**PROTEINURIA:** The new 2024 KDIGO Clinical Practice Guideline for the Evaluation and Management of Chronic Kidney Disease suggests replacing the use of the term proteinuria by the abovementioned categories of albuminuria. Category A3 reflects what was considered proteinuria [1].

**MODE OF INHERITANCE:** This is the manner in which a genetic trait or disorder is passed from one generation to the next. Alport syndrome caused by pathogenic variants in *COL4A5* is inherited in an X-linked manner (XLAS). Alport syndrome caused by pathogenic variants in *COL4A3* or *COL4A4* can be inherited in an autosomal recessive (ARAS – in case of a biallalic gene defect) or autosomal dominant manner (ADAS – in case of a presence of a single monoallelic (heterozygous) P/LP variant). Risk to family members depends on mode of inheritance ranging from 100% in case of daughters of XLAS males, 50% for children of both genders in case of XLAS females and ADAS individuals, 25% for siblings of ARAS individuals and 0% in case of sons of XLAS males. For details refer to: <https://www.ncbi.nlm.nih.gov/books/NBK1207/#alport.Genetic_Counseling>

**(LIKELY) PATHOGENIC VARIANT (P/LP)**: A pathogenic variant is a change in the DNA sequence of a gene that causes a person to have or be at risk of developing a certain genetic disorder or disease.

The term 'likely pathogenic' applies to a change having >90% certainty of the variant being disease-causing, but below a higher “pathogenic” threshold.

**VARIANT OF UNCERTAIN SIGNIFICANCE (VUS)**: A change in a gene’s DNA sequence that has an unknown effect on a person’s health. There is usually not enough information about a variant of uncertain significance to know whether it increases or decreases a person’s risk of developing a disease.

**PHASE OF THE VARIANT** (cis, trans): Phase refers to the genetic relationship between a pair of variants; that is, whether the variants are on the same copy of the chromosome (cis) or on two different copies of the chromosome (trans).

**DIGENIC INHERITANCE:** This refers to variants at two loci that explain the phenotype better than a variant at a single locus. In case of Alport spectrum disorders, “digenic” disease refers to simultaneous presence of two pathogenic variants in different *COL4A3/4/5* genes[2].

***Appendix 2: Support for people living with Alport syndrome***

**Table of support available for people living with Alport syndrome**

| **Local kidney unit** | Your local kidney unit can be contacted anytime. You can get information, support from staff and your questions answered that relate to your individual care. Some units have experts who can help with diet, psychological support and social services. |
| --- | --- |
| **National organizations supporting people living with Alport and Alport syndrome** | These organisations vary in size and focus from country to country. They have huge amount of resources, news and support available for people living with Alport syndrome, carers, doctors and researchers. They can answer your questions and give you information, local support groups, clinical trials, treatments, social events, information days and fundraising. See their websites and social media:  **Australia**: Alport Foundation of Australia – [www.alport.org.au](http://www.alport.org.au), contact email: [info@alport.org.au](mailto:info@alport.org.au)  **Belgium:** AIRG Belgique - airg-belgique.org  **China**: Chinese Alport Syndrome Parents Organisation – WeChat, contact email: [hello@henizaiyigi.com](mailto:hello@henizaiyigi.com)  **Germany**: Alport Selbsthilfe - [www.alport-selbsthilfe.de](http://www.alport-selbsthilfe.de), contact email: [Vorstand@Alport-Selbsthilfe.de](mailto:Vorstand@Alport-Selbsthilfe.de)  **France**: AIRG France - [www.airg-france.fr](http://www.airg-france.fr), contact email: [airg.permanence@orange.fr](mailto:airg.permanence@orange.fr)  **Israel**: Alport Foundation Israel - facebook  **Italy**: A.S.A.L., Associazione Sindrome di Alport - www.alport.it, contact email: [informazioni.asal@gmail.com](mailto:informazioni.asal@gmail.com)  **Macedonia:** contact email: [gordana_david@yahoo.com](mailto:gordana_david@yahoo.com)  **Spain:** AIRG España – [www.airg-e.org](http://www.airg-e.org), contact email: [info@airg-e.org](mailto:info@airg-e.org)  **Switzerland:** AIRG Suisse - www.airg-suisse.org, contact email: info@airg-suisse.org  **UK**: alport UK - alportuk.org, contact email: [info@alportuk.org](mailto:info@alportuk.org), follow @alportuk  **USA:** Alport Syndrome Foundation - alportsyndrome.org, contact email: info@alportsyndrome.org, follow @alportsyndromefndn  **The Netherlands**: Nierpatiënten Vereniging Nederland (NVN) – www.[nvn.nl](mailto:nvn.nl), contact email: secretariaat@nvn.nl  **Other countries without a national patient group:** contact **Alport Syndrome Alliance** – alportsyndromealliance.org, contact email: workshops@alportsyndromealliance.org |
| **Alport Warriors** | A closed Facebook group which is moderated by alport UK – families and individuals from all over the country and internationally connect to share the key questions and issues they face every day living with Alport and Alport syndrome. There is always someone with practical tips and experience to share. See how student Joseph McLean lives with Alport syndrome: [https://youtu.be/4bz5-tK6m6w](https://eur03.safelinks.protection.outlook.com/?url=https%3A%2F%2Fyoutu.be%2F4bz5-tK6m6w&data=05%7C01%7CHolly.Mabillard2%40newcastle.ac.uk%7C64544ef476b24842b47e08db55eefecd%7C9c5012c9b61644c2a91766814fbe3e87%7C1%7C0%7C638198257177585531%7CUnknown%7CTWFpbGZsb3d8eyJWIjoiMC4wLjAwMDAiLCJQIjoiV2luMzIiLCJBTiI6Ik1haWwiLCJXVCI6Mn0%3D%7C3000%7C%7C%7C&sdata=aqIjYOw%2FAR8lNeYwj467P6nZBdHLNp8UH5cWZjPmt94%3D&reserved=0) |
| **Alport Avengers** | A group of young adults (18-35 years old) living with Alport syndrome who provide each other with help around the key time that Alport and Alport syndrome impacts their lives most. They help each other practically and thoughtfully through tough times and meet socially on a regular basis. Talking about their condition with their peers is sometimes the only time they feel ‘normal’. |
| **Alport social weekends and Alport information days** | Check out the events in a specific country by going to the websites for the specific country listed above. Events are hosted to engage those who are newly diagnosed and connect them with others in similar situations and hear about the latest research. People living with Alport syndrome organise the events with the local clinical teams and make sure all age groups get their questions answered and have fun and get to know the cities they visit. Young patients in Manchester, UK share their experiences: <https://www.youtube.com/watch?v=77p7nzKz6nc> |
| **International Experts** | International experts are available to explain the complexities of Alport and Alport syndrome - for both clinicians and people living with Alport and Alport syndrome |
| **Peer Support** | A chat on the phone with someone who knows what it feels like to live with Alport or Alport syndrome can be lifechanging. Connect to local or national services for grants, psychosocial support or someone nearby. |
| **International workshops on Alport Syndrome** | Get involved in research and join an international workshop on Alport syndrome. For those interested in science, research and travel, Alport UK organizes a unique programme of international workshops that bring together people across the world the **Alport Syndrome Alliance** – a global network of inspiring people living with Alport and Alport syndrome, clinicians and researchers developing new treatments and knowledge. As part of a wider mental health programme, we encourage young adults to travel abroad and build their confidence to ask questions about their condition and contribute to research discussions. Highlights from Siena workshop in Italy: [https://youtu.be/QH8mDTmKaVU](https://eur03.safelinks.protection.outlook.com/?url=https%3A%2F%2Fyoutu.be%2FQH8mDTmKaVU&data=05%7C01%7CHolly.Mabillard2%40newcastle.ac.uk%7C64544ef476b24842b47e08db55eefecd%7C9c5012c9b61644c2a91766814fbe3e87%7C1%7C0%7C638198257177741826%7CUnknown%7CTWFpbGZsb3d8eyJWIjoiMC4wLjAwMDAiLCJQIjoiV2luMzIiLCJBTiI6Ik1haWwiLCJXVCI6Mn0%3D%7C3000%7C%7C%7C&sdata=qcXqxFimm28pxUU8IqRlj2asFGZSY3Qi2Cesb65u4nY%3D&reserved=0" \o "https://eur03.safelinks.protection.outlook.com/?url=https%3A%2F%2Fyoutu.be%2FQH8mDTmKaVU&data=05%7C01%7CHolly.Mabillard2%40newcastle.ac.uk%7C64544ef476b24842b47e08db55eefecd%7C9c5012c9b61644c2a91766814fbe3e87%7C1%7C0%7C638198257177741826%7CUnknown%7CTWFpbGZsb3d8eyJWIjoiMC4wLjAwMDAiLCJQIjoiV2luMzIiLCJBTiI6Ik1haWwiLCJXVCI6Mn0%3D%7C3000%7C%7C%7C&sdata=qcXqxFimm28pxUU8IqRlj2asFGZSY3Qi2Cesb65u4nY%3D&reserved=0) |
| **Health Talk** | This website is a hub where thousands of people have shared their experiences on film of what it is like to have a health condition in addition to explanations from experts. There are many on Alport and Alport syndrome. See website <https://healthtalk.org> (see Alport syndrome stories) |

**References**

1. KDIGO 2024 Clinical Practice Guideline for the Evaluation and Management of Chronic Kidney Disease. *Kidney Int* 2024;**105**:S117-s314.

2. Savige J, Renieri A, Ars E et al. Digenic Alport Syndrome. *Clin J Am Soc Nephrol* 2022;**17**:1697-706.
